# Optimizing Tongue Fluid Sampling and Testing Protocols for Enhanced PRRSV Isolation from Perinatal Swine Mortalities

**DOI:** 10.3390/v17010102

**Published:** 2025-01-14

**Authors:** Onyekachukwu Henry Osemeke, Isadora Machado, Elisa De Conti, Mariah Musskopf, Mafalda Pedro Mil-Homens, Samuel Stutzman, Baoqing Guo, Thomas Petznick, Gustavo De-Sousa-E Silva, Phillip Gauger, Jianqiang Zhang, Daniel C. L. Linhares

**Affiliations:** 1 Veterinary Diagnostic and Production Animal Medicine, College of Veterinary Medicine, Iowa State University, Ames, IA 50011-3619, USAbqguo@iastate.edu (B.G.); gustavos@iastate.edu (G.D.-S.-E.S.); pcgauger@iastate.edu (P.G.); 2 Ronnfeldt Farms, Lyons, NE 68038-4574, USA

**Keywords:** PRRSV, postmortem tissues, tongue fluids, PCR, virus isolation, surveillance

## Abstract

Porcine reproductive and respiratory syndrome virus (PRRSV) remains a major concern for swine health. Isolating PRRSV is essential for identifying infectious viruses and for vaccine formulation. This study evaluated the potential of using tongue fluid (TF) from perinatal piglet mortalities for PRRSV isolation. Four collection protocols were tested: extracting TF from fresh tissues using phosphate-buffered saline (PBS group), extracting TF from fresh tissues using virus transportation medium (VTM group), extracting TF from freeze-thawed tissue (freeze-thaw group), and using tissue homogenates (homogenate group). Two cell lines (ZMAC and MARC-145) and primary alveolar macrophages (PAM) were evaluated for their effect on successful PRRSV isolation. An eligible PRRSV-positive unstable breeding herd in Midwestern USA was chosen for the study. Tongues were collected in 20 batches (~30 mortalities per batch). Within each batch, each tongue tissue was cut into four quarters, with each quarter randomly assigned to one of the four collection protocols and RT-qPCR tested. Virus isolation (VI) was attempted on 10 batches. The mean RT-qPCR cycle threshold (Ct) values for the PBS, VTM, freeze-thaw, and homogenate groups were 21.9, 21.8, 22.6, and 24.8, respectively. The VI success rate was 22.6%, 12.1%, 2.8%, and 2.8% in the PBS, VTM, freeze-thaw, and homogenate groups, respectively. The probability of successful VI was 3.1% and 21.0% in the MARC-145 and ZMAC cell lines, respectively, and 4.8% in the PAM cells. TF from perinatal mortalities is an option for PRRS VI, aiding in PRRSV monitoring and control programs.

## 1. Introduction

Porcine reproductive and respiratory syndrome virus (PRRSV) surveillance is particularly challenging in low-prevalence scenarios due to the cost of testing representative units and the complexities associated with PRRSV ecology [1]. Recently, the postmortem tongue or tongue fluid (TF) sample was demonstrated to be a cost-effective and population-sensitive approach for PRRSV surveillance in swine herds. TF samples are collected by aggregating sections of tongues from stillborn or dead piglets, typically in a sterile bag. These tissues, or the fluids they release, are then submitted to diagnostic laboratories for tests [2,3].

Although reverse transcription-quantitative polymerase chain reaction (RT-qPCR) detection of PRRSV RNA is the most requested test for PRRSV surveillance in the US [4], isolating the live virus remains crucial for confirming the presence of infectious PRRSV in a herd. The isolation of PRRSV from tissues, including those of stillborn piglets, has been previously demonstrated [5,6]. However, there is currently no documented evidence of live PRRSV being isolated specifically from TF samples. Our research team had made unsuccessful attempts previously to obtain live PRRSV isolates from TF samples submitted from the field (unpublished). We hypothesize that this may be due to the low quality of viral particles in decomposing postmortem tissues [6,7,8].

Various cells are used for the in vitro isolation and propagation of PRRSV [9,10,11]. Among these, MARC-145 cells, derived from African green monkey kidney cells, are widely used due to their proven ability to support the replication of PRRSV-1 and PRRSV-2. These cells are easy to culture and yield high virus titers, making them a convenient choice for laboratory studies. Porcine alveolar macrophages (PAM), as primary cells from the lungs of pigs, provide an authentic model for isolating PRRSV and studying PRRSV-host interactions [12]. Additionally, ZMAC cells, derived from porcine macrophages, serve as a valuable tool for isolating and characterizing PRRSV, further enhancing the range of in vitro models available for research and laboratory testing [11,13,14].

Transportation media are used to preserve the diagnostic quality of the samples. Cell culture medium is a nutrient-rich option that maintains virus viability during transport, though its cost can be prohibitive for routine use [15]. Phosphate-buffered saline (PBS), a more affordable alternative, is often used because it preserves tissue integrity and prevents cell lysis. PBS is particularly effective for short-term transport if samples are quickly processed in the lab [16]. Alternatively, unprocessed tissues can be transported without a medium and frozen shortly after collection to minimize viral degradation [17].

This study aimed to address the challenges associated with isolating live PRRSV from TF samples. By refining both collection and testing protocols, we aimed to overcome the issues of potentially low viral quality in decomposing tissues and, consequently, improve the diagnostic utility of TF samples for PRRSV surveillance. Strengthening these methods could provide a cost-effective, herd-representative approach to detecting and isolating PRRSV, even in low-prevalence herds where surveillance is particularly challenging. In addition, this work may help clarify the relationship between PCR-based detection of PRRSV and the presence of infectious virus, offering swine practitioners valuable insights for live vaccine formulation and more effective disease management strategies.

## 2. Materials and Methods

### 2.1. Study Population and Samples

The study was conducted on a 5000-head sow farm in Midwestern USA that had recently experienced a PRRSV outbreak, with ≥2 stillborn piglets per litter reported in the week prior to sampling. Tongue tissues, each approximately three inches long, were collected from all piglet mortalities within 24 h of birth, including stillborn piglets, naturally deceased piglets, and those euthanized due to poor condition. Every group of 30 perinatal mortalities made up one batch. Each tongue within each batch was divided into four sections, and each section was placed in sterile Ziploc bags, resulting in four Ziploc bags containing tongue quarters from 30 piglets. Each of the four Ziploc bags was randomly allocated to one of the four collection protocols (or treatment groups) being evaluated. This was repeated until 20 batches of perinatal mortalities were processed, as described above. Of the 20 batches of perinatal mortalities, three comprised stillbirths exclusively, while the remaining 17 batches were mixes of stillbirths, naturally deceased piglets, and those euthanized due to poor condition. Even though all 20 batches were sent for PRRSV RT-qPCR testing across all groups (i.e., 80 samples), only 10 batches (i.e., 40 samples) were submitted for viral isolation. A sample size of 40 provides >80% statistical power to detect a statistical difference in the proportion of successful virus isolations between the 4 (DF = 3) collection protocols, assuming a type 1 error rate of 10% and an effect size of 0.5.

### 2.2. TF Collection Protocols

**Figure 1:**
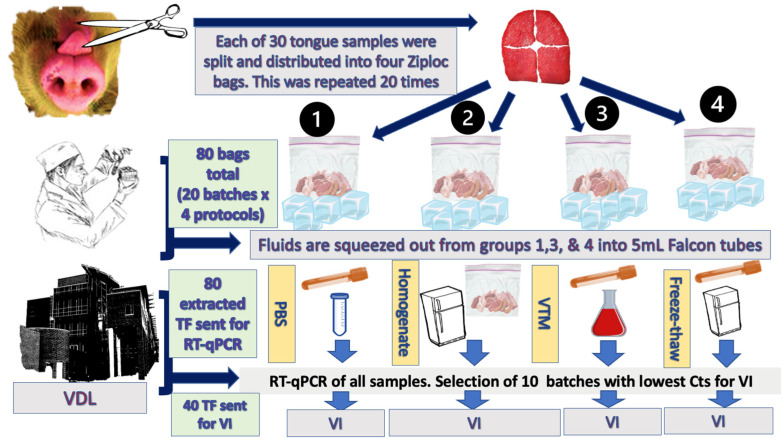
The workflow for the study. Tongue samples from each batch of 30 stillborn or dead piglets were divided into four sections, each allocated to one protocol: PBS (4 mL PBS added, wrung, and stored at −70 °C), Homogenate (homogenized in Earle’s Balanced Salt Solution, centrifuged, and stored at −70 °C), VTM (4 mL virus transport medium added, wrung, and stored at −70 °C), and Freeze-Thaw (frozen at −20 °C, thawed, mixed with PBS, and stored at −70 °C). All 80 samples underwent RT-qPCR testing, with the 10 batches yielding the lowest Ct values submitted for viral isolation (VI).

To extract TF from the aggregated tongue quarters in the Ziploc bags, the following protocols were employed (Figure 1):

(1) PBS Group: For this group, 4 mL of PBS was added to the Ziploc bag, and the contents were then wrung into a 5 mL conical tube (Corning Science Mexico S.A. de C.V., Tamaulipas, Mexico). The tube was kept on dry ice until it reached the veterinary diagnostic laboratory (VDL), where it was stored at −70 °C until testing.
(2) Homogenate Group: For this group, the Ziploc bag and its contents were kept on ice until arrival at the VDL. Tissues were processed in 1× Earle’s Balanced Salt Solution (pH 7.2–7.4) (Sigma-Aldrich, MO, USA) to generate roughly 10–20% (g/volume) homogenates using a Geno grinder instrument following the standard procedures (e.g., at tissue setting for 2 min at 1500 rpm). Subsequently, the homogenates were centrifuged at 1400× *g* for 5 min at 4 °C. The supernatants were transferred to new tubes and stored at −70 °C until testing.
(3) VTM Group: For this group, 4 mL of virus transport medium was added to the bag, and the contents were wrung into a 5 mL conical tube. The tube was kept on dry ice until arrival at the VDL. This sample was stored at −70 °C until testing. The virus transport medium was a mixture of RPMI 1640 medium supplemented with 100 mg/mL streptomycin and 0.25 mg/mL amphotericin.
(4) Freeze-Thaw Group: For this group, tongue tissues were frozen at −20 °C and then thawed after 24 h; 4 mL of PBS was added to each bag, and the contents were wrung into a 5 mL conical tube. This sample was kept at −70 °C until testing.

### 2.3. RT-qPCR and Virus Isolation

All 80 samples (20 batches × 4 collection protocols) were processed, and aliquots were submitted to the Iowa State University Veterinary Diagnostic Laboratory for PRRSV RT-qPCR testing. Briefly, nucleic acids were extracted from 100 µL of samples using a MagMAX Pathogen RNA/DNA Kit (Thermo Fisher Scientific, Waltham, MA, USA) and a Kingfisher Flex instrument (Thermo Fisher Scientific) and eluted into 90 µL of elution buffer. A commercial screening real-time RT-PCR, VetMAX^TM^ PRRSV NA&EU 3.0 Kit (Thermo Fisher Scientific), was used to test the presence of PRRSV RNA following the manufacturer’s instructions. This PRRSV screening PCR targets the conserved genomic regions and can simultaneously detect and differentiate between PRRSV-1 and PRRSV-2. For either PRRSV-1 or PRRSV-2, the threshold cycle (CT) < 37 was considered positive, and CT ≥ 37 was considered negative.

Based on the lowest mean cycle threshold (Ct) values, the 10 batches with the highest potential for viral presence were selected for virus isolation. Three types of cell culture were used for virus isolation: MARC-145 cell line, derived from African monkey kidney cells; ZMAC cell line, derived from porcine lung lavage fluid; and a batch of primary alveolar macrophages (PAM), purchased from Rural Technologies, Inc. (Brookings, SD, USA). The samples were filtered through 0.22 µm syringe filters (MilliporeSigma, Burlington, MA, USA), and 300 µL of the filtered samples were inoculated into each type of cell (MARC-145, ZMAC, and PAM) cultured in 24-well plates. After 1 h of incubation with ZMAC and PAM, or 2 h of incubation with MARC-145 at 37 °C with 5% CO_2_, the inoculum was removed and 2 mL of fresh culture medium was added. The plates were incubated at 37 °C with 5% CO_2_ and cytopathic effects (CPE) were checked daily. The virus isolation results were confirmed by immunofluorescence staining using PRRSV nucleocapsid protein-specific monoclonal antibody conjugates, as described previously [11].

### 2.4. ORF5 Sequencing

The ORF5 sequences of PRRSV present in the 10 batches of TF used for VI attempts and the VI-positive isolates were determined using the Sanger method, following the previously described procedures [14]. The restriction fragment length polymorphism (RFLP) patterns based on three restriction enzymes, *Mlu*I, *Hinc*II, and *Sac*II, on PRRSV-2 ORF5 sequences were determined as previously described [18]. ORF5 nucleotide identities between different PRRSV-2 sequences were calculated using the MegAlign 17 program in the DNASTAR Lasergene 17 software. Multiple sequence alignment was performed using the progressive method (FFT-NS-2) in MAFFT v7.407. The phylogenetic tree from the multiple sequence alignment result was constructed using maximum likelihood and 1000 bootstrap replicates in IQ-Tree v2.2.2.6. ORF5-based genetic lineages and sublineages were determined following the recently revised genetic classification system [19].

### 2.5. Statistical Analysis

Descriptive statistical analyses were performed to summarize and visualize the distribution of Ct values across the different TF collection protocols. A simple linear regression model was used to assess the association between TF collection protocols (predictor) and Ct values (outcome). The least-squares mean Ct values for each protocol were estimated from the regression model, with pairwise comparisons conducted using Sidak’s adjustment to control the family-wise error rate.

The factors influencing the probability of successful virus isolation (VI) were evaluated using a multivariable approach to account for potential confounding effects among predictor variables and to isolate the independent influence of each factor on the likelihood of PRRSV isolation. A generalized linear model (GLM) with a binomial error distribution and a logit link function was used to model the relationship between the predictor variables and the probability of VI. In this model, the response variable was binary (PRRSV isolation: yes or no), while the predictor variables included cell line, collection protocol, and mortality category. The least-squares mean probability estimates for each predictor variable were obtained from the logistic regression model using a post hoc test with Sidak’s adjustment to control the family-wise error rate across multiple sets of pairwise comparisons.

Graphical plots were generated to visually present the results of the regression model for each predictor variable. A type 1 error rate of 10% was used for the statistical analyses. All statistical analyses were conducted using the R statistical software version 4.4.1 [20].

## 3. Results

### 3.1. RT-qPCR Outcomes by Collection Protocol

**Figure 2:**
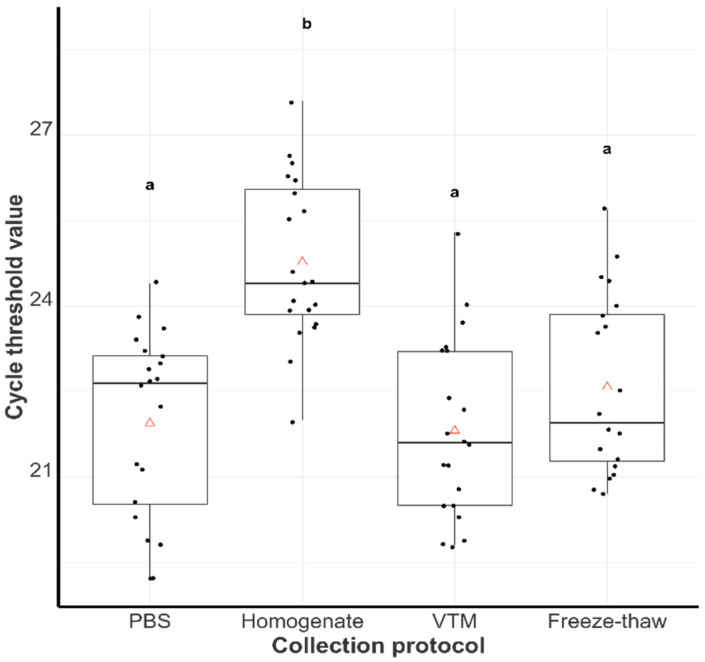
Distribution of cycle threshold values for the phosphate-buffered saline (PBS) group, tissue homogenate group (Homogenate), virus transport medium (VTM) group, and freeze-thaw (Freeze-thaw) group. Groups with different letters are statistically different (*p* < 0.1). The central triangles within the boxplots represent the mean Ct value for the collection protocol.

All 80 samples tested positive for PRRSV using RT-qPCR, with the cycle threshold (Ct) values ranging from 19.2 to 27.6. The PBS and VTM groups had comparable mean Ct values of 21.9 and 21.8, respectively, while the freeze-thaw group had a mean Ct value of 22.6. There were no statistically significant differences (*p* ≥ 0.01) in the mean Ct values of these groups. In contrast, the homogenate group exhibited the highest mean Ct value of 24.8, significantly different from the mean Ct values of the other groups (Figure 2).

### 3.2. Virus Isolation Outcomes

Five of the ten batches used for VI attempts showed at least one positive VI result in one of the experimental groups and in at least one cell type. Batches 1, 5, and 11 (Table 1) consisted exclusively of stillborn piglets, whereas the remaining batches included a mix of stillborn, deceased, and euthanized piglets.

#### 3.2.1. Probability of Successful PRRSV Isolation by Collection Protocol

**Figure 3:**
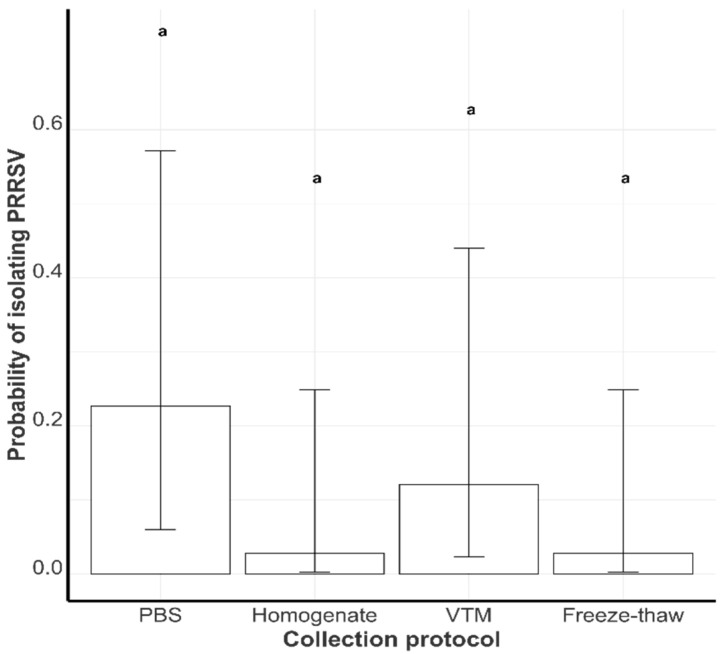
The least-square mean probability of live PRRSV isolation in the phosphate-buffered saline (PBS), tissue homogenate (Homogenate), virus transport medium (VTM), and freeze-thaw groups. Groups with different letters are statistically different (*p* < 0.1).

The virus isolation success rates were 22.6% (8.1%, 49.1%), 12.1% (3.4%, 39.1%), 2.8% (0.4%, 16.4%), and 2.8% (0.4%, 16.4%) in the PBS, VTM, freeze-thaw, and homogenate groups (Figure 3). These probabilities were, however, not statistically different (*p* > 0.1) after adjusting for all pairwise comparisons.

#### 3.2.2. Probability of Successful PRRSV Isolation by Cell Type

**Figure 4:**
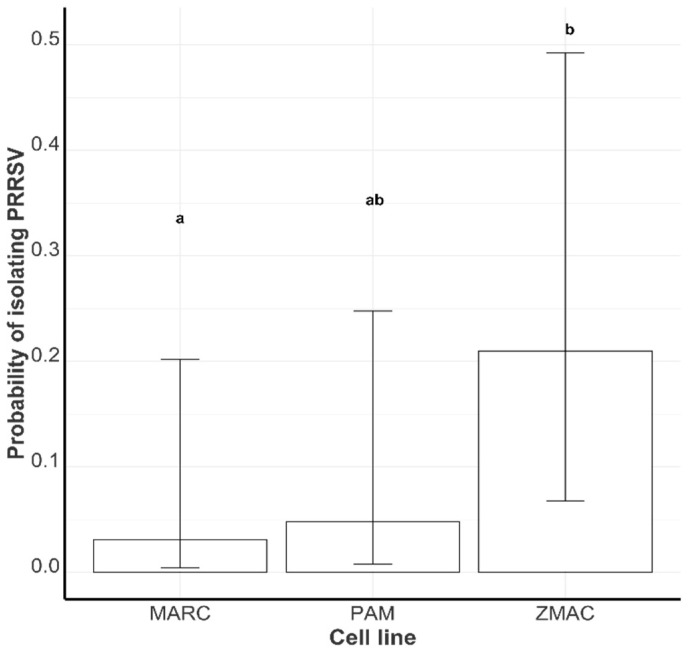
The least-square mean probability of live PRRSV isolation in the MARC-145 cell line (MARC), PAM cell line (PAM), and ZMAC cell line (ZMAC). Groups with different letters are statistically different (*p* < 0.1).

The virus isolation success rates in the MARC-145 and ZMAC cell lines were 3.1% (0.59%, 14.9%) and 21.0% (8.4, 43.5%), respectively, and 4.8% (1.1%, 19.1%) in PAM cells (Figure 4). The VI success rate in the ZMAC cell line was statistically higher than the success rate in the MARC-145 cell line; all other pairwise comparisons were not statistically different (Figure 4).

#### 3.2.3. Probability of Successful PRRSV Isolation by Mortality Category

**Figure 5:**
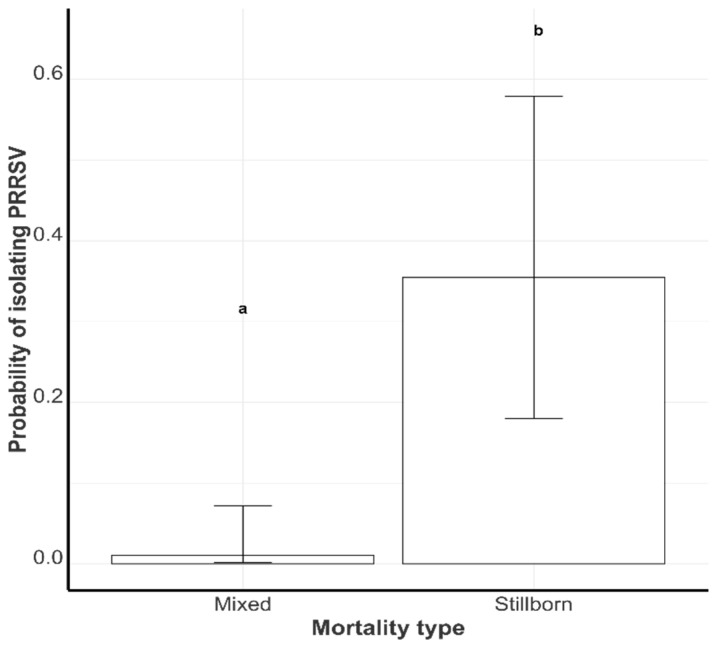
The least-square mean probability of live PRRSV isolation in the mixed pig batches (Mixed) and stillborn pig batches (Stillborn). Groups with different letters are statistically different (*p* < 0.1).

The virus isolation success rate in perinatal mortality batches that were exclusively stillborn piglets was 35.45% (17.97%, 57.92%), while the success rate in the mixed batches was 1.03% (0.14%, 7.16%). These VI success rates were statistically different (Figure 5).

### 3.3. ORF5 Sequence Analysis of PRRSV in TF Used for Virus Isolation

**Figure 6:**
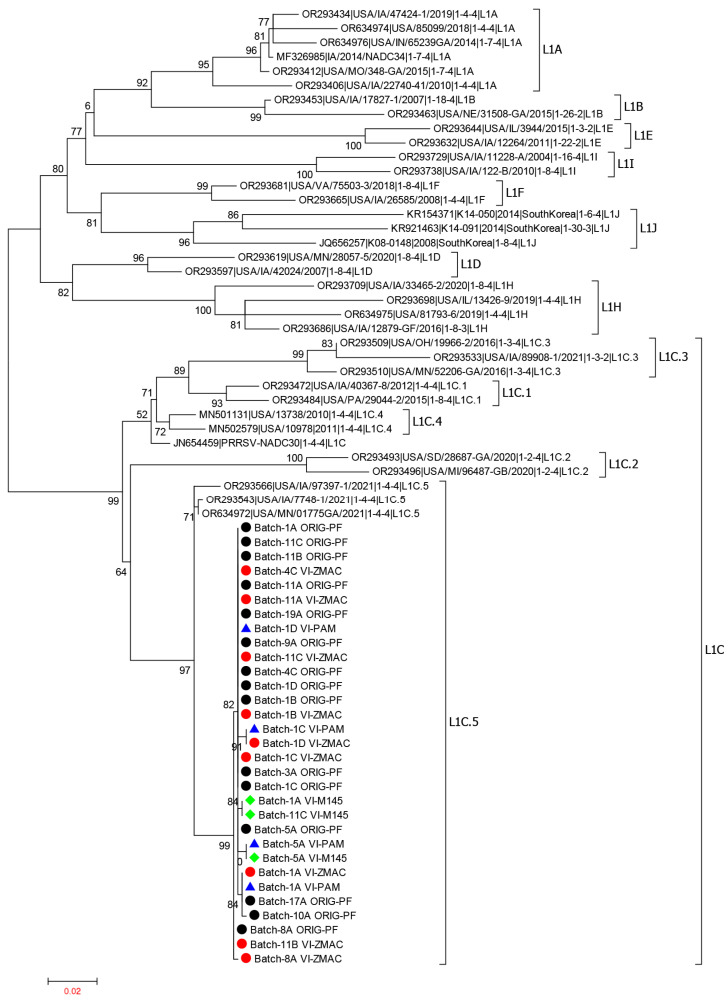
Phylogenetic tree based on ORF5 nucleotides of PRRSV-2 lineage 1 sequences. The representative L1A, L1B, L1C (LC.1, LC.2, L1C.3, L1C.4, L1C.5, and L1C-unclade), L1D, L1E, L1F, L1H, L1I, and L1J are depicted. The PRRSV-2 ORF5 sequences determined in this study are shown using solid bullet points (black circles for sequences in the original processing fluid samples, red circles for sequences in the ZMAC isolates; blue triangles for sequences in PAM isolates, and green diamonds for sequences in MARC-145 isolates). The letters A, B, C, and D after the “Batch-” represent extracting TF from fresh tissues using phosphate-buffered saline (PBS group), using tissue homogenates (homogenate group), extracting TF from fresh tissues using virus transportation medium (VTM group), and extracting TF from freeze-thawed tissue (freeze-thaw group), respectively.

Based on the ORF5 sequence analysis, all 10 batches of TF used for VI attempts contained PRRS viruses belonging to the L1C.5 variant (Figure 6) with 99–100% nucleotide identity to each other, although they belonged to the RFLP patterns 1-3-4, 1-4-4, or 1-3-2 (Table 1). For VI-positive samples, the virus isolates (regardless of the cell types) had sequences matching (99–100% nucleotide identity) to the virus present in the corresponding clinical samples.

## 4. Discussion

This study highlights the potential of using TF from perinatal mortalities for effective PRRSV isolation (VI). Aggregate samples, such as TF, provide a more comprehensive representation of PRRSV strains within a herd as they capture fluid contributions from multiple animals that were likely deceased due to PRRSV. This not only strengthens surveillance, but also provides herd-representative PRRSV isolates suitable for whole-genome sequencing or preparing stock solutions for live virus inoculation.

This study primarily assessed different TF collection protocols and virus isolation cell types for the successful recovery of live PRRSV from perinatal mortalities. Specifically, four TF collection and handling protocols were compared: extracting TF from freshly collected tissues using phosphate-buffered saline (PBS group), extracting TF from freshly collected tissues using virus transportation medium (VTM group), extracting TF from freeze-thawed tissue (freeze-thaw group), and using tongue tissue homogenates (homogenate group). Two protocols (extracting TF from freeze-thawed tissues and extracting TF through tissue homogenization) reflected common practices used by practitioners, while the other two collection and handling protocols (extracting TF from fresh mortalities using PBS or VTM with immediate storage on dry ice) followed what we evaluated as better-practice sampling methods for virus isolation.

Results showed that the PBS, VTM, and freeze-thaw groups had statistically lower mean Ct values compared to the homogenate group, indicating relatively higher virus titers within the three groups. The probability of successful VI was lowest in the homogenate and freeze-thaw groups. Considering the RT-qPCR and VI results together, the homogenate group performed the most poorly. We hypothesize that the homogenization process might lead to a loss of viral integrity or a dilution effect that reduces the chances of detecting live viruses. In addition, it may be impractical to take tissue snippets from all tongue tissues within a submission for tissue homogenization. Hence, tissue homogenates may not have as many animals represented within them as the originally submitted aggregate sample.

Regarding the cell lines used for PRRSV isolation, ZMAC cells yielded the highest probability of successful VI, consistent with previous studies highlighting their effectiveness for PRRSV replication [11]. MARC-145 cells, though commonly used, showed a lower VI success probability, indicating that they may not be as receptive to PRRSV as porcine tissue-derived cells and cell lines. Porcine alveolar macrophages (PAMs) had a lower VI probability than ZMAC cells. This outcome, though unexpected, could be attributed to the inherent variability of primary cells, such as PAMs, which are more prone to batch-to-batch differences, contamination, and degradation. In contrast, immortalized cell lines like MARC-145 and ZMAC offer greater consistency and stability. Moreover, the handling and maintenance of primary cells require more stringent protocols, potentially complicating the virus isolation process and reducing efficiency.

In the current study, all TF samples were collected from one farm. ORF5 sequencing confirmed that the virus present in the 10 batches of TF used for VI all belonged to the L1C.5 variant and were genetically similar to each other. This ensures that the VI outcome differences in a certain cell type are attributable to sample collection and processing differences rather than virus strain differences. However, the VI differences observed between ZMAC, PAMs, and MARC-145 cells should be interpreted cautiously because only isolation of the L1C.5 virus in different cell types was evaluated in this study. A previous study demonstrated that PRRSV genetic lineage can impact the virus isolation success rate in different cell lines; for example, PRRSV-2 in genetic lineages 1 and 8 was isolated more successfully in ZMAC cells than in MARC-145 cells, whereas PRRSV-2 in lineage 5 was isolated in ZMAC and MARC-145 cells with similar success rates [11]. In future studies, TF samples containing another PRRSV strain (e.g., lineage 5) should be collected under different protocols and then evaluated for VI in different cell types for comparison. For this study, there was a limited sample size for VI (n = 10 batches), which constrained our ability to conduct more robust statistical modeling, such as incorporating possible interaction between predictors. The results from this study revealed that TF batches consisting solely of stillborn piglets had a significantly higher probability of successful VI than mixed batches of stillborn, dead, and euthanized piglets. The higher success rate of virus isolation from stillborn piglets compared to other categories of mortalities may stem from several factors. Notably, stillborn piglets have not suckled and, therefore, have not ingested colostrum, which contains maternal antibodies and immune cells that could potentially interfere with virus isolation [21]. The absence of these immune components may have allowed for more effective recovery of live viruses. Additionally, it is possible that stillborn piglets have relatively higher viral loads from intrauterine infection, providing a more concentrated source of PRRSV for isolation [22]. This finding further underscores the importance of selecting appropriate (risk-based) sample sources for PRRSV surveillance.

Overall, the extraction of TF from freshly collected tissues using phosphate-buffered saline (PBS group) was the most effective TF collection protocol, with its performance not statistically different from TF extraction from freshly collected tissues using virus transportation medium (VTM group). The ZMAC cell line outperformed the PAM cells and the MARC-145 cell line under the conditions of this study. Perinatal batches comprised solely of stillborn piglets were more likely to have a positive PRRSV isolation test. Ensuring timely collection and proper storage of samples enhances PRRSV isolation from TF. Future research should address the generalizability of these findings across different farm conditions and PRRSV strains and evaluate the long-term stability of TF samples under various storage conditions.

## 5. Conclusions

Live PRRSV can be successfully isolated from TF. Extracting TF from fresh stillborn piglets using PBS or VTM enhances the likelihood of successful virus isolation. In this study, the ZMAC cell line demonstrated superior performance compared to the MARC-145 cell line and PAM cells for PRRSV isolation. Maintaining a strict cold chain from sample collection to laboratory arrival is critical for preserving the diagnostic integrity of the samples.

Virus isolation from such aggregate samples enables the efficient co-detection of multiple PRRSV strains within a herd, if present. This approach enhances surveillance efforts and facilitates obtaining representative isolates for further testing or the production of live vaccines.

## Figures and Tables

**Table 1:** Comparison of PRRSV isolation success across postmortem tongue fluid (TF) collection and handling protocols, mortality categories, and cells.

Serial Number	Pig Batch Number	Mortality Category	Experimental Group	RT-qPCR Ct Value	RFLP and ORF5-Based Lineage	ZMAC	PAM	MARC-145
1	1	Stillborn	PBS	19.2	1-3-4 L1C.5	Positive	Positive	Positive
2	1	Stillborn	Homogenate	22.0	1-3-4 L1C.5	Positive	-	-
3	1	Stillborn	VTM	19.8	1-3-4 L1C.5	Positive	Positive	-
4	1	Stillborn	Freeze-thaw	21.0	1-3-4 L1C.5	Positive	Positive	-
5	3	Mix	PBS	20.6	1-3-4 L1C.5	-	-	-
6	3	Mix	Homogenate	24.6	1-3-4 L1C.5	-	-	-
7	3	Mix	VTM	20.5	1-3-4 L1C.5	-	-	-
8	3	Mix	Freeze-thaw	21.8		-	-	-
9	4	Mix	PBS	19.8	1-3-4 L1C.5	-	-	-
10	4	Mix	Homogenate	23.0	1-3-4 L1C.5	-	-	-
11	4	Mix	VTM	20.3	1-3-4 L1C.5	Positive	-	-
12	4	Mix	Freeze-thaw	21.8	1-3-4 L1C.5	-	-	-
13	5	Stillborn	PBS	23.0	1-3-2 L1C.5	-	Positive	Positive
14	5	Stillborn	Homogenate	23.5	1-3-2 L1C.5	-	-	-
15	5	Stillborn	VTM	19.9	1-3-2 L1C.5	-	-	-
16	5	Stillborn	Freeze-thaw	21.3	1-3-2 L1C.5	-	-	-
17	8	Mix	PBS	21.2	1-4-4 L1C.5	Positive	-	-
18	8	Mix	Homogenate	23.9	1-4-4 L1C.5	-	-	-
19	8	Mix	VTM	21.2	1-4-4 L1C.5	-	-	-
20	8	Mix	Freeze-thaw	22.5	1-4-4 L1C.5	-	-	-
21	9	Mix	PBS	22.2	1-3-4 L1C.5	-	-	-
22	9	Mix	Homogenate	24.4	1-3-4 L1C.5	-	-	-
23	9	Mix	VTM	21.2	1-3-4 L1C.5	-	-	-
24	9	Mix	Freeze-thaw	21.5	1-3-4 L1C.5	-	-	-
25	10	Mix	PBS	19.2	1-3-4 L1C.5	-	-	-
26	10	Mix	Homogenate	24.0	1-3-4 L1C.5	-	-	-
27	10	Mix	VTM	20.8	1-3-4 L1C.5	-	-	-
28	10	Mix	Freeze-thaw	20.7	1-3-4 L1C.5	-	-	-
29	11	Stillborn	PBS	20.3	1-3-4 L1C.5	Positive	-	-
30	11	Stillborn	Homogenate	23.6	1-3-4 L1C.5	Positive	-	-
31	11	Stillborn	VTM	21.6	1-3-4 L1C.5	Positive	-	Positive
32	11	Stillborn	Freeze-thaw	21.0	1-3-4 L1C.5	-	-	-
33	17	Mix	PBS	19.9	1-3-4 L1C.5	-	-	-
34	17	Mix	Homogenate	23.9	1-3-4 L1C.5	-	-	-
35	17	Mix	VTM	20.5	1-3-4 L1C.5	-	-	-
36	17	Mix	Freeze-thaw	20.8	1-3-4 L1C.5	-	-	-
37	19	Mix	PBS	21.1	1-3-4 L1C.5	-	-	-
38	19	Mix	Homogenate	23.7	1-3-4 L1C.5	-	-	-
39	19	Mix	VTM	19.8	1-3-4 L1C.5	-	-	-
40	19	Mix	Freeze-thaw	21.2	1-3-4 L1C.5	-	-	-

Mortality categories include Stillborn (only stillborn piglets) and Mix (a combination of stillborn, dead, and euthanized piglets). Experimental groups represent different sample preparation and handling protocols: extracting TF from fresh tissues using phosphate-buffered saline (PBS group), extracting TF from fresh tissues using virus transportation medium (VTM group), extracting TF from freeze-thawed tissue (freeze-thaw group), and using tissue homogenates (homogenate group). RFLP and ORF5 lineage subtypes (e.g., 1-3-4 L1C.5) identify PRRSV genotypes. Virus isolation success is indicated for ZMAC (porcine alveolar macrophages), PAM (primary porcine alveolar macrophages), and MARC-145 (monkey kidney cell line). “Positive” denotes successful isolation, and “-” denotes failure.

## Data Availability

Any additional data not herein present will be provided upon request.
